# Subtle changes in *Plasmodium falciparum* infection complexity following enhanced intervention in Malawi

**DOI:** 10.1016/j.actatropica.2014.11.008

**Published:** 2015-02

**Authors:** Tamika J. Sisya, Raphael M. Kamn’gona, Jimmy A. Vareta, Joseph M. Fulakeza, Mavuto F.J. Mukaka, Karl B. Seydel, Miriam K. Laufer, Terrie E. Taylor, Standwell C. Nkhoma

**Affiliations:** a Malawi-Liverpool-Wellcome Trust Clinical Research Programme, University of Malawi College of Medicine, Blantyre, Malawi; b Blantyre Malaria Project, University of Malawi College of Medicine, Blantyre, Malawi; c Department of International Health, Bloomberg School of Public Health, Johns Hopkins University, Baltimore, MD, USA; d Osteopathic Medical Specialties, College of Osteopathic Medicine, Michigan State University, East Lansing, MI, USA; e Center for Vaccine Development, University of Maryland School of Medicine, Baltimore, MD, USA; f Liverpool School of Tropical Medicine, Pembroke Place, L3 5QA Liverpool, UK

**Keywords:** *Plasmodium falciparum*, Malaria control interventions, Multiple-genotype infections, Genetic diversity, Effective population size, Genetic differentiation

## Abstract

With support from the Global Fund, the United States President's Malaria Initiative (PMI) and other cooperating partners, Malawi is implementing a comprehensive malaria control programme involving indoor residual spraying in targeted districts, universal coverage with insecticide-treated bed nets, use of rapid diagnostic tests to confirm the clinical diagnosis of malaria and use of the highly effective artemisinin-based combination therapy, artemether-lumefantrine (AL), as the first-line treatment for malaria. We genotyped 24 genome-wide single nucleotide polymorphisms (SNPs) in *Plasmodium falciparum* infections (*n* = 316) sampled from a single location in Malawi before (2006 and 2007) and after enhanced intervention (2008 and 2012). The SNP data generated were used to examine temporal changes in the proportion of multiple-genotype infections (MIs), mean number of heterozygous SNPs within MIs, parasite genetic diversity (expected heterozygosity and genotypic richness), multilocus linkage disequilibrium and effective population size (*N*_*e*_). While the proportion of MIs, expected heterozygosity, genotypic richness, multilocus linkage disequilibrium and *N*_*e*_ were unchanged over time, the mean number (±standard deviation) of heterozygous SNPs within MIs decreased significantly (*p* = 0.01) from 9(±1) in 2006 to 7(±1) in 2012. These findings indicate that the genetic diversity of *P. falciparum* malaria parasites in this area remains high, suggesting that only subtle gains, if any, have been made in reducing malaria transmission. Continued surveillance is required to evaluate the impact of malaria control interventions in this area and the rest of Malawi, and to better target control interventions.

## Highlights

• We examined impact of intense malaria control on parasite genetic structure in Malawi.
• Malaria infections sampled before and after intense control were genotyped at 24 SNPs.
• Despite intense control efforts, parasite genetic diversity was unchanged over time.
• Only the mean number of heterozygous SNPs within infections showed change over time.
• Findings suggest minimal or no change in malaria transmission despite intense control.

## Graphical abstract

**Figure:**
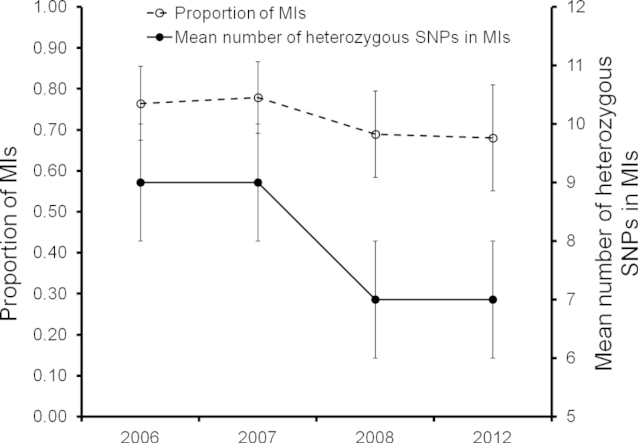

## 1 Introduction

Malaria-endemic countries are rapidly scaling up malaria control interventions to reduce the burden of malaria. This has led to significant reductions in the numbers of malaria cases and malaria-related deaths in several countries (Chizema-Kawesha et al., 2010; O’meara et al., 2010; World Health Organization, 2013a, 2013b). Since 2007, the Malawi government has been implementing a comprehensive malaria control programme involving indoor residual spraying in seven targeted districts (excluding Blantyre), switch from targeted use of insecticide-treated bed nets (ITNs) as a means of preventing malaria among children and pregnant women attending EPI (Expanded Programme on Immunization) and antenatal clinics to universal ITN coverage, and change from sulphadoxine-pyrimethamine (SP) to an artemisinin-based combination therapy, artemether-lumefantrine (AL), as the first-line treatment for malaria. In addition, since 2011, rapid diagnostic tests have been deployed at all health facilities to confirm the clinical diagnosis of malaria. This programme is being implemented with support from the Global Fund, the United States President's Malaria Initiative (PMI) and other cooperating partners. However, there are limited and conflicting data on the impact of this programme. While some reports indicate a significant decrease in malaria burden in response to enhanced intervention in Malawi (Feng et al., 2010; Mathanga et al., 2005; World Health Organization, 2013a), others show no effect (Roca-Feltrer et al., 2012; Bennett et al., 2013; Okiro et al., 2013). We might expect to see significant reductions in malaria transmission partly because, unlike SP, which has weak activity against transmissible stages of the parasite (gametocytes), AL is highly active against both asexual parasites and gametocytes (Okell et al., 2008; Sutherland et al., 2005). Its potency against gametocytes could directly reduce malaria transmission in this setting, and lead to detectable changes in *P. falciparum* genetic structure.

Previous studies have demonstrated a strong correlation between malaria transmission intensity, genotypic complexity of infections and parasite genetic structure (Conway et al., 1999; Anderson et al., 2000; Zhong et al., 2007; Daniels et al., 2013; Nkhoma et al., 2013). In regions of intense transmission, *Plasmodium falciparum* exhibits a highly outbred population structure characterized by extensive outcrossing, high incidence of multiple-genotype infections (MIs), low levels of linkage disequilibrium, and a low prevalence of genetically identical infections (Conway et al., 1999; Anderson et al., 2000; Zhong et al., 2007). In contrast, in low transmission regions, *P. falciparum* shows a predominantly clonal (inbred) population structure characterized by high levels of self-fertilization, limited recombination, few MIs, high levels of linkage disequilibrium and a high incidence of genetically identical infections (Conway et al., 1999; Anderson et al., 2000; Zhong et al., 2007). In addition, *P. falciparum* populations from low transmission regions show reduced levels of genetic diversity and low effective population sizes. Most of these patterns are also observed in longitudinal studies of malaria parasites in areas where transmission is declining due to intense control efforts (Daniels et al., 2013; Nkhoma et al., 2013). In such areas, the incidence of MIs wanes, the proportion of patients bearing identical parasite genotypes increases, multilocus linkage disequilibrium increases and the mean number of heterozygous SNPs within MIs (an indirect measure of the genotypic complexity of infections) decreases (Daniels et al., 2013; Nkhoma et al., 2013). These observations support the practice of using parasite genetic measures as proxies for changes in malaria transmission following enhanced intervention.

The central goal of this study was to examine *P. falciparum* genetic structure at a single location in Malawi over time to identify changes which might correspond to the effects of various interventions on malaria transmission.

## 2 Materials and methods

### 2.1 Collection of malaria parasite samples

Malaria-infected blood samples were collected from 316 children less than 5 years old presenting to Ndirande Health Centre in Blantyre with uncomplicated *P. falciparum* malaria before (2006 and 2007) and after (2008 and 2012) the intensification of control efforts. Ndirande Health Centre is the only public health facility in Ndirande Township, and serves a catchment area with a population of ∼300,000 people. In recent years, this area and the rest of Malawi have seen increased uptake of malaria control interventions including increased use of ITNs, introduction of rapid diagnostic tests to confirm the clinical diagnosis of malaria and switch to a more efficacious drug, AL, as the first-line treatment for malaria. To our knowledge, the population in this area does not have easy access to other drugs, and AL is available through prescription only. We employed a passive case detection system to identify children with uncomplicated *P. falciparum* malaria amongst children seeking care at the health centre. No special effort was made to find unsuspected or asymptomatic disease in the community where the enrolled children lived. At least 46 malaria-infected blood samples were sampled per year. This sample size allows the detection of a 30% difference in the proportion of MIs before and after enhanced intervention with 80% power and 95% confidence assuming a pre-intervention prevalence of 78%. During the sampling of 2006–2008 infections, we avoided sampling members of the same household and serial sampling of the same person by carefully matching records of patients already enrolled into the study with those of prospective study participants. We did this to preclude parasite relatedness structure driven by similar host genetic background. However, when collecting malaria-infected blood samples in 2012, no special effort was made to avoid sampling members of the same household or serial sampling of the same individual. All samples were collected with the approval of the child's parent or guardian under the auspices of antimalarial drug resistance studies and health facility-based cross-sectional malaria surveys approved by the University of Malawi College of Medicine Research and Ethics Committee.

### 2.2 Parasite DNA extraction

Samples from 2006 to 2008 were in the form of whole blood, and were extracted for parasite DNA using Qiagen DNA Mini Kits (Qiagen, UK). Samples from 2012 were in the form of filter paper blood spots and were extracted using a two-step protocol to maximize parasite DNA yield. Blood was first eluted from filter paper blood spots using Gensolve Kits (Integen X, USA), followed by parasite DNA extraction with 96-well QIAamp DNA Blood Kits (Qiagen, UK) (Nkhoma et al., 2013). The concentration of parasite DNA in each sample was determined using the NanoDrop 1000 Spectrophotometer.

### 2.3 SNP genotyping

We genotyped all parasite infections at 24 polymorphic single nucleotide polymorphisms (SNPs) using the molecular barcode assay as described previously (Daniels et al., 2008). The names and positions of SNPs genotyped were as described in the original methodology paper (Daniels et al., 2008) and as annotated in PlasmoDB version 5.0. Parasite DNA samples from MR4 *P. falciparum* laboratory strains 3D7, V1S, W2-8E, DD2, HP710 and an artificial mixture of W2-8E and 3D7 were included in each genotyping run as positive controls. W2-8E is a parasite clone isolated from W2 by dilution cloning while HP710 is a clone from the Thai-Myanmar border. To perform the assay, a master mix consisting of 2.95 μl of commercial grade nuclease-free water, 0.05 μl of the 40x Taqman SNP assay and 5 μl of the TaqMan Universal PCR Master Mix (Applied Biosystems Catalogue # 4364343) per reaction was added to each well of a 96-well real-time PCR plate (Applied Biosystems Catalogue # 4346906) pre-loaded with 2 μl (10 ng) of each parasite DNA sample. The concentration of parasite DNA in the template was standardized across all samples to avoid the potential technical bias of scoring more heterozygous loci in samples with high parasite densities. Parasite DNA samples were amplified on the StepOne real-time PCR instrument (Applied Biosystems), followed by analysis of the results with Applied Biosystem's proprietary Allelic Discrimination software. Where the software did not give genotype calls directly, we made allele calls manually by examining both the amplification plot and the multi-component plot. To examine if the 24-SNP molecular barcode assay has enough resolution power, we also genotyped six parasite clones isolated from a multiple-genotype infection sampled at Ndirande Health Centre in 2008. These clones were previously genotyped using a custom-made 384-SNP GoldenGate assay, and shown to be either closely related (differing only at a few of the 316 successfully genotyped SNPs) or genetically identical (sharing alleles at all the 316 successfully genotyped SNPs) (Nkhoma et al., 2012).

### 2.4 Identification of multiple-genotype infections

We identified multiple-genotype infections by examining the number of heterozygous SNPs in each infection genotyped. Because human blood-stage malaria parasites are haploid, a single-genotype infection is expected to have only one allele at each SNP locus while a multiple-genotype infection is expected to carry multiple alleles. However, in this study, we used a minimum of two heterozygous SNPs as a threshold for classifying MIs because one random SNP out of the 24 SNPs genotyped was occasionally wrongly scored as heterozygous even in well-characterised monoclonal infections. SNP data for single-genotype infections were used to estimate SNP allele frequencies, and to measure temporal changes in *P. falciparum* genetic diversity indices (expected heterozygosity and genotypic richness), multilocus linkage disequilibrium, effective population size, proportion of identical parasite infections, and to examine if there was significant genetic differentiation between parasite samples collected before and after the intensification of malaria control efforts. SNP data for multiple-genotype infections (MIs) were used to examine temporal changes in the proportion of MIs and the mean number of heterozygous SNPs within MIs. We conducted analyses both by the year of sampling and by pooling samples from early (2006 and 2007) and late (2008 and 2012) sampling periods.

### 2.5 Resolving relationships among parasite genotypes

We computed the proportion of SNP alleles shared (*ps*) between all pairwise comparisons of single-genotype infections and clustered infections on the UPGMA tree based on the genetic distance metric, 1-*ps*, using PHYLIP (Felsenstein, 1993). Parasite genotypes were considered “unique” if they differed at one or more of the 24 SNPs genotyped and “identical” if they shared alleles at all the 24 SNPs. Identical parasite genotypes arise when a clonal parasite lineage passes through mosquitoes unchanged due to self-fertilization and is repeatedly sampled from multiple human hosts. We examined the proportion of patients infected with identical parasite genotypes over time to infer changes in self-fertilization rates in this parasite population. This was achieved by calculating the percentage of patients infected with identical parasite genotypes amongst the single-genotype infections that we had identified.

### 2.6 Genetic diversity and population structure

We used the genotypic richness index, *R* (Dorken and Eckert, 2001) and Nei's expected heterozygosity (*H*_*e*_) index (Nei, 1978) to examine changes in parasite genetic diversity over time. *R* measures the proportion of unique genotypes present in the samples and is estimated as: *R* = (*G* − *1*)/(*N* − *1*) where *G* is the number of unique genotypes and *N* is the sample size (Dorken and Eckert, 2001). In addition, we investigated whether or not there was significant genetic differentiation between parasites sampled before and after enhanced intervention. We did this to examine whether or not the parasite population had become more subdivided due to the pronounced effect of genetic drift and migration in declining populations. Genetic differentiation was expressed as the pairwise fixation (F_ST_) index (Weir and Cockerham, 1984), estimated using FSTAT (Goudet, 1995).

### 2.7 Multilocus linkage disequilibrium

We compared multilocus LD in parasite infections collected in 2006, 2007, 2008 and 2012 to seek evidence of increased LD over time. Multilocus LD was measured using the statistic I_A_S (standardized index of association), which compares the observed variance in the numbers of alleles shared between parasites with that expected when parasites share no alleles at different loci (Haubold and Hudson, 2000). I_A_S estimations were performed with and without identical parasite genotypes because we expect identical parasite genotypes to be the major source of LD in these infections (Nkhoma et al., 2013).

### 2.8 Effective population size

We examined whether or not *N*_*e*_ had decreased following enhanced intervention. We used fluctuations in SNP allele frequencies between adjacent years to estimate the variance effective population size (*N*_*e*_*V*). *N*_*e*_*V* was estimated using the pseudo-maximum likelihood method implemented in the programme, MLNE v. 1547 (Wang, 2001) and the moments-based temporal methods of Nei/Tajima (Nei and Tajima, 1981), Pollak (Pollak, 1983) and Jorde/Ryman (Jorde and Ryman, 2007) implemented in NeEstimator v. 2.0 (Do et al., 2013). When estimating *N*_*e*_, we assumed that *P. falciparum* has a generation time of 2 months (Nkhoma et al., 2013), and we allowed a maximum *N*_*e*_ value of 10,000.

### 2.9 Statistical tests

Proportions were compared using the Chi-square test while means were compared using the Two sample *t*-test. Means and proportions are reported as the mean ± standard deviation. Figures in square brackets reported for the genotypic richness index and effective population size are 95% confidence intervals. The degree of genetic differentiation between temporally spaced samples was assessed using the Wilcoxon signed-rank test. The Kruskal–Wallis rank-sum test was used to examine whether or not there was significant variation in parasite density over time. Univariate Poisson regression was used to model the rate of decline in parasite density over time. In addition, we used univariate negative binomial regression to examine associations between the number of heterozygous SNPs within MIs and each of the following covariates: log-transformed parasite density, patient age, sex, season and year of sampling. Negative binomial regression was preferred over Poisson regression wherever there was over-dispersion with the Poisson model (i.e. the variance in the mean number of heterozygous SNPs within MIs being substantially higher than the mean). This is because in such a case, negative binomial regression provides a better fit than the Poisson model. All tests were implemented in STATA version 10.1 (College Station, TX, USA).

## 3 Results

### 3.1 Data summary

We successfully genotyped 295 out of 316 parasite infections sampled from children less than 5 years old presenting to Ndirande Health Centre with uncomplicated *P. falciparum* malaria before and after enhanced intervention. Summary statistics for parasite sampling and basic genetic data are presented in Table 1. Out of the 24 SNPs genotyped, only the G/T SNP on chromosome 14 at position 00755729 of the *P. falciparum* genome (PlasmoDB version 5.0) was monomorphic in all the four temporal samples (Supplementary information, Table S1). The rest were polymorphic with minor allele frequencies ranging from 0.03 to 0.50 (Supplementary information, Table S1). Of the 295 parasite infections that we successfully genotyped, 217 were classified as multiple-genotype infections (MIs) while the remaining 78 were single-genotype infections.

Supplementary Table S1 related to this article can be found, in the online version, at http://dx.doi.org/10.1016/j.actatropica.2014.11.008.

Supplementary Table S1 Sample details and 24-SNP genotype data for parasite isolates analysed. Each parasite isolate was genotyped at 24 SNPs using the molecular barcode assay (Daniels et al., 2008). The name of each SNP consists of the chromosome on which it is found and its position on the chromosome as annotated in PlasmoDB version 5.0. Letters “A”, “C”, “G” or “T” highlighted in yellow below SNP names represent minor alleles. Figures highlighted in green are minor allele frequencies as estimated from single-genotype infections only. SNP 24 on chromosome 14 position 000755729 of the *P. falciparum* genome, PlasmoDB version 5.0, was monomorphic in all parasite isolates analysed (allele frequency of the “T” allele = 0) just like in a previous study, which examined parasite genetic diversity in malaria parasite infections from Blantyre (Milner et al., 2012). Genotyping controls are shown in rows with red highlighting. Multilocus genotype refers to the number assigned to each parasite haplotype (i.e. a set of alleles at the 24 genotyped SNPs in single-genotype infections). Genetically identical infections are assigned the same number while genetically distinguishable infections are assigned unique numbers. Clonality denotes the genetic complexity of an infection i.e. whether the infection contains multiple parasite genotypes (M) or a single parasite genotype (S). N/A = not applicable and ND = not determined.

### 3.2 Relationships among parasite infections

**Fig. 1:**
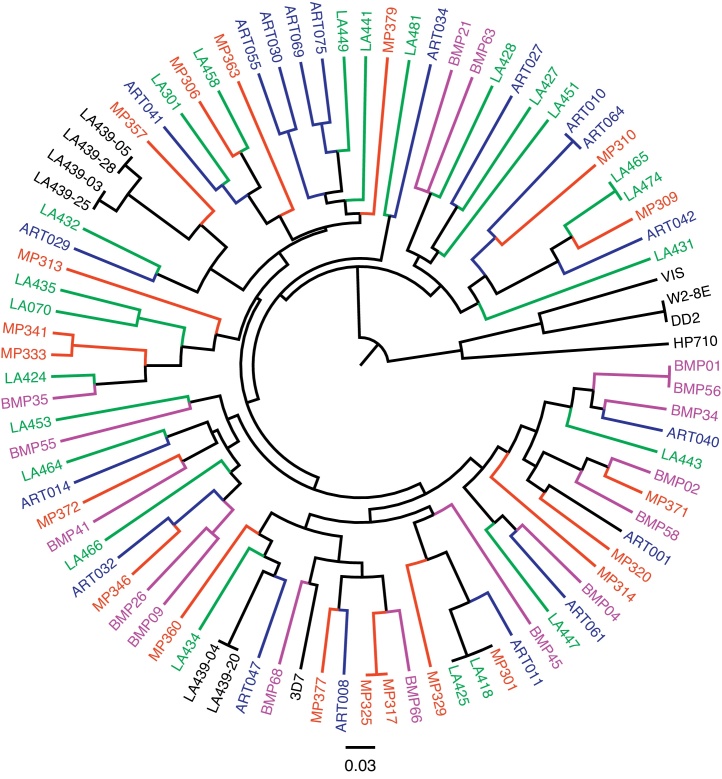
UPGMA tree showing relationships amongst 78 single-genotype infections and genotyping controls. The tree is constructed from a pairwise distance matrix, 1-*ps*, where *ps* is the proportion of SNP alleles shared between any two parasite isolates. Single-genotype infections sampled during each of the 4 years are coded using different colours (red = 2006, blue = 2007, green = 2008, purple = 2012) while laboratory controls are coded in black. Laboratory controls LA439-05, LA439-28, LA439-03, LA439-25, LA439-04 and LA439-20 are parasite clones isolated from a multiple-genotype infection sampled from Ndirande health centre in 2008. These were previously identified to be genetically identical using the 384-SNP assay (Nkhoma et al., 2012). LA439-05 and LA439-28, LA439-03 and LA439-25, LA439-04 and LA439-20 cluster together on the tree and are identical at all 24 SNPs genotyped. This result provides reassurance that the 24-SNP assay has enough resolution power for identifying both unique and identical parasite genotypes. Patients MP301, LA418 and LA425 had identical barcodes. Similarly, patients MP317 and MP325, ART010 and ART064, LA465 and LA474 had the same barcodes, indicating that they were infected with identical parasite genotypes.

Relationships amongst single-genotype infections sampled and laboratory controls are shown on the UPGMA tree (Fig. 1). As expected, isogenic laboratory controls W2-8E and DD2 cluster together on the tree akin to closely related parasite clones isolated from a multiple-genotype infection sampled at Ndirande Health Centre in 2008. Parasite clones deemed to be genetically identical using the 384-SNP GoldenGate assay shared alleles at all the 24 SNPs genotyped (Fig. 1). In addition, parasite clones that differed at one SNP using the 384-SNP GoldenGate assay (Nkhoma et al., 2012) were found to differ at just one SNP using the 24-SNP molecular barcode assay. Using the molecular barcode assay, we identified 73 parasite genotypes infecting at least one patient over the whole sampling period (Fig. 1). While most genotypes were detected only once, five (7%) were sampled multiple times. The proportion of patients infected with identical parasite genotypes remained the same between 2006 and 2012 (10% in 2006 vs 13% in 2012; *p* = 0.813). Patients infected with identical parasite genotypes were from different households. There was no evidence of serial sampling of identical parasite genotypes from the same individual.

### 3.3 Genetic composition of multiple-genotype infections

**Fig. 2:**
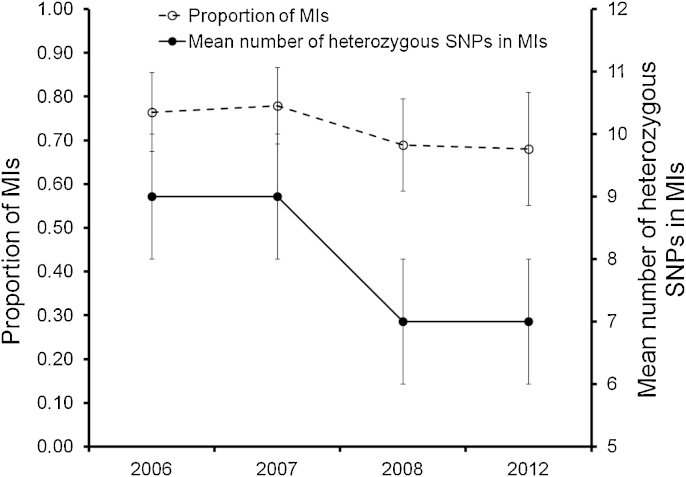
Change in the composition of multiple-genotype infections over time. We examined changes in the frequency of multiple-genotype infections (MIs) and the mean number of heterozygous SNPs within MIs over the time frame observed. Error bars are 95% confidence intervals. While the frequency of MIs decreased only slightly and non-significantly (*p* > 0.05), the mean number of heterozygous SNPs within MIs decreased significantly over time (*p* < 0.05).

We observed subtle changes in the composition of multiple-genotype infections over the time frame observed (Fig. 2). The incidence of MIs decreased non-significantly from 0.76 ± 0.42 in 2006 to 0.68 ± 0.47 in 2012 (*p* = 0.823). In contrast, the average number of heterozygous SNPs within MIs decreased significantly from 9 ± 1 in 2006 to 7 ± 1 in 2012 (*p* = 0.01).

### 3.4 Lack of change in parasite genetic diversity

We observed no significant temporal change in parasite genetic diversity as expressed by the expected heterozygosity index, *H*_*e*_ and the genotypic richness index, *R* (Table 1). The mean *H*_*e*_ for parasite samples collected in 2006 (*H*_*e*_ = 0.37 ± 0.16) did not differ significantly (*p* = 0.34) from that of 2012 samples (*H*_*e*_ = 0.32 ± 0.17). Similarly, the mean *H*_*e*_ for samples from both 2006 and 2007 was 0.38 ± 0.15 compared with 0.37 ± 0.16 in samples from both 2008 and 2012 (*p* = 0.58). Similarly, *R* was 0.95 [0.74–1.00] in 2006 compared with 0.93 [0.68–1.00] in 2012 (*p* = 0.81).

### 3.5 Absence of temporal genetic differentiation and multilocus linkage disequilibrium

There was no significant genetic differentiation between parasite samples collected in 2006 and 2012 (*F*_ST_ = 0.030 ± 0.036), 2007 and 2012 (*F*_ST_ = 0.038 ± 0.043), 2007 and 2008 (*F*_ST_ = −0.013 ± 0.018), and between pooled samples from 2006 to 2007 compared with samples collected in 2008 and 2012 combined (*F*_ST_ = 0.008 ± 0.015; *p* = 0.355). Similarly, there was no significant multilocus linkage disequilibrium (*p* > 0.05) in all temporal samples.

### 3.6 Lack of change in effective population size of *P. falciparum*

All the four measures of the variance effective population size showed no significant change (*p* > 0.05) over time (Table 2).

## 4 Discussion

The primary goal of this study was to genotype *P. falciparum* malaria infections sampled from a single location in Malawi before and after enhanced intervention to examine if various population genetic parameters respond in a manner expected of declining parasite transmission. We wanted to see if there were detectable changes in parasite genetic structure and infection complexity consistent with a decrease in malaria transmission. While most population genetic proxies of malaria transmission were unchanged over time, the mean number of heterozygous SNPs within MIs showed significant change. This change was accompanied by significant variation in parasite density over time (Kruskal–Wallis rank-sum test: *χ*^2^ = 13.364, *p* = 0.004). Poisson regression analysis revealed an average of 2% decline in parasite density per year (incidence rate ratio, IRR = 0.9847 [0.9841–0.9853] 95% CI; *p* < 0.001). However, univariate negative binomial regression analysis revealed that the number of heterozygous SNPs within MIs was not associated with parasite density (*p* = 0.062), season (*p* = 0.056), patient age (*p* = 0.903) or sex (*p* = 0.979). The only variable associated with a reduction in the number of heterozygous SNPs within MIs is the year of sampling. The lack of association between the number of heterozygous SNPs within infections and parasite density suggests that the observed temporal decrease in the mean number of heterozygous SNPs within MIs was not simply a result of sampling low density infections. The mean number of heterozygous SNPs within MIs can provide an indirect measure of the number or relatedness of parasite genotypes within MIs (Nkhoma et al., 2013). Therefore, the observed decrease in the mean number of heterozygous SNPs suggests that MIs from 2008 and 2012, on average, contained fewer or significantly more related parasite genotypes than MIs from 2006 to 2007. This decrease most likely arose from serial transmission of MIs between human hosts due to reduced transmission. We attribute this subtle change in malaria transmission to the 2007 drug policy switch from SP to AL, increased ITN use over the time frame observed, and to a lesser extent, to the introduction of rapid diagnostic tests as a confirmatory tool for the clinical diagnosis of malaria in 2011. While there are no area-specific intervention coverage data, country-wide data indicate that household ownership of at least one ITN increased from 38% in 2006 to 55% in 2012 (World Health Organization, 2013a). Similarly, the proportion of children who slept under an ITN the night preceding the survey increased from 25% in 2006 to 55% in 2012 (World Health Organization, 2013a). Our finding that only the mean number of heterozygous SNPs within MIs showed significant change over time suggests that compared with other genetic parameters examined, this measure has a relatively high sensitivity and can detect minor reductions in transmission intensity. This measure may be far more informative in high transmission areas undergoing a transition to low transmission than in low transmission regions.

**Supplementary Fig. S1:**
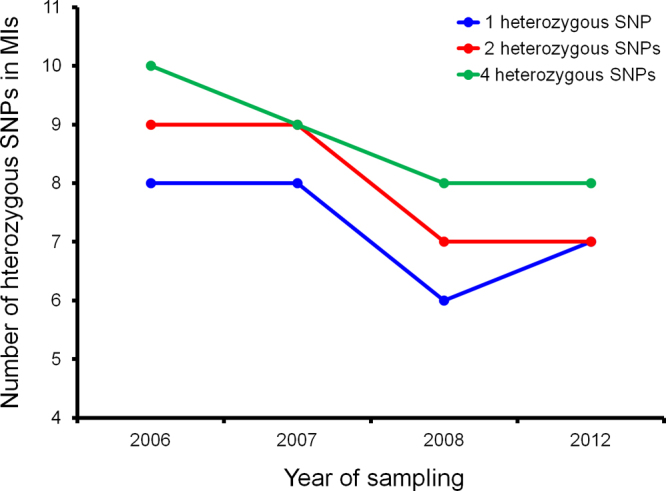
Change in the mean number of heterozygous SNPs within multiple-genotype infections (MIs) is independent of the threshold used for calling MIs. We examined whether the use of different thresholds (≥1, ≥2 or ≥4 heterozygous SNPs) for categorizing infections as either single-genotype or multiple-genotype infections would have an effect on the association observed between the number of heterozygous SNPs within infections and sampling year. Regardless of whether ≥1, ≥2 or ≥4 heterozygous SNPs were used as the cut-off for defining multiple-genotype infections (MIs), the mean number of heterozygous SNPs within MIs was found to decrease significantly over time (*p* < 0.05).

Our finding that the frequency of MIs, the proportion of patients infected with identical parasite genotypes, multilocus linkage disequilibrium and genotypic richness did not change significantly following enhanced intervention suggests no change in parasite selfing rate. It points to very little or no reduction in malaria transmission in this area, and is consistent with some of the available epidemiological data (Roca-Feltrer et al., 2012; Bennett et al., 2013; Okiro et al., 2013). However, the lack of temporal change in the proportion of MIs in this setting contrasts previous observations on the Thai-Myanmar border and Senegal where enhanced malaria control was accompanied by a significant decrease in the frequency of MIs (Nkhoma et al., 2013; Daniels et al., 2013). It is possible that in areas of intense malaria transmission such as Malawi where MIs are extremely common, the proportion of MIs is less sensitive to intense malaria control efforts. The frequency of MIs takes much longer to decline following the intensification of control efforts, and is preceded by a significant decrease in the mean number of heterozygous SNPs within MIs. The proportion of MIs estimated by this study was slightly higher (68–78%) than in a previous study (50%) from the same setting (Milner et al., 2012). This may be due to the fact that most of the infections analysed in the previous study were severe; these tend to be genotypically less complex than the uncomplicated malaria infections (Milner et al., 2012) characterized in this study. This discrepancy may also be due to differences in the thresholds used for calling MIs. In this study, infections with ≥2 heterozygous SNPs were classified as MIs while in the Milner et al. study, infections were classified as MIs if they had ≥4 heterozygous SNPs. Therefore, the threshold used in this study would slightly underestimate the proportion of single-genotype infections and overestimate the proportion of multiple-genotype infections for the same dataset. Nevertheless, regardless of the thresholds used for calling MIs, the mean number of heterozygous SNPs within MIs was found to decrease significantly over time (Supplementary information, Fig. S1). The lack of significant genetic differentiation between parasite populations sampled before and after enhanced intervention indicates that both populations were panmictic. Two reasons might explain the lack of change in most population genetic parameters despite enhanced control efforts. It is possible that there is truly no change or very minimal change in malaria transmission despite enhanced intervention. It is also possible that our study lacks the power to detect a change in transmission.

Supplementary Fig. S1 related to this article can be found, in the online version, at http://dx.doi.org/10.1016/j.actatropica.2014.11.008.

The lack of detectable change in *N*_*e*_ and *H*_*e*_ mirrors previous observations on the Thai-Myanmar border (Nkhoma et al., 2013) and in Sri Lanka (Gunawardena et al., 2014) where a marked decline in malaria transmission did not lead to a significant decrease in both *N*_*e*_ and *H*_*e*_. Why do these parameters not change? It is well known that in order to have a substantial effect on *H*_*e*_, a bottleneck must be either narrow (very low *N*_*e*_ for a few generations) or very prolonged (moderate *N*_*e*_ for many generations). Therefore, we may have failed to detect changes in *N*_*e*_ and *H*_*e*_ because the drop in transmission is not large enough or has not been sustained for long enough to significantly alter *N*_*e*_ and *H*_*e*_. In addition, declining populations of small organisms like *P. falciparum* tend to have relatively large population sizes, which are less sensitive to the loss of genetic diversity. It is possible that not enough of the parasite population was sampled to allow accurate estimation of these parameters since our power calculations were based on detecting change in the proportion of MIs. With respect to *N*_*e*_, Palstra and Ruzzante (2008) suggest that at least 10% of the population's effective size should be sampled. This would mean sampling at least 1000 infections per year in this setting. Nonetheless, even if sampling effort is increased to reduce sampling variance, allele frequency changes may be too small to detect in large populations (Hare et al., 2011; Neafsey, 2013). The population size would need to drop by several orders of magnitude before any short term effect on *H*_*e*_ and *N*_*e*_ is detectable. Given the exponential relationship between *N*_*e*_ and genetic drift, it is perhaps overly optimistic to expect *N*_*e*_ to change over the time frame observed (1–5 years). The *N*_*e*_ metric is very sensitive to *N*_*e*_ decreasing from 10 to 1 or 100 to 10 but detecting the change from 10,000 to 1000 or even 1000 down to 100, is very difficult (Neafsey, 2013). We suggest that changes in *N*_*e*_ based on allele frequency variance may only be detectable and informative in parasite populations at pre-elimination and elimination stages: malaria transmission in Malawi is still too high and robust to be impacted in terms of this variable.

In conclusion, results from this study indicate that the genetic structure of malaria parasites in Ndirande, Blantyre, has largely remained the same over 5 years despite the rapid scale up of malaria control interventions including the switch from SP to AL as the first-line treatment for malaria. All population genetic parameters examined, except the mean number of heterozygous SNPs within MIs, were unchanged 1–5 years after enhanced intervention. We conclude that any reduction in malaria transmission left most population genetic parameters unchanged. It appears that different population genetic parameters are informative in various windows of the transmission spectrum. It is most likely that serial transmission of MIs previously founded by superinfection predominates during early stages of transmission reduction. This results in increased within-host relatedness of parasites, and is manifested by reduced numbers of heterozygous SNPs in MIs. The fact that only the mean number of heterozygous SNPs in MIs shows change suggests that this parameter has a higher sensitivity than other metrics for monitoring changes in transmission levels, and can detect minor reductions in malaria transmission intensity. The lack of change in key population genetic parameters including *H*_*e*_ and *N*_*e*_ indicates that only subtle gains, if any, have been made in reducing transmission. Therefore, continued surveillance using epidemiological, entomological and population genetic approaches will be required to evaluate the impact of control interventions on malaria transmission in the study area and the rest of Malawi, and to better target malaria control interventions. We believe that genetic monitoring of malaria parasite populations will play a central role in identifying areas where parasite genetic diversity remains high despite a significant decrease in the number of malaria cases. Targeted interventions could be deployed in such areas to dramatically reduce parasite genetic diversity and prevent resurgence of malaria.

## Financial support

This study was supported by a Research Initiative Award (grant # IA10) from the Malaria Capacity Development Consortium (MCDC) and a Wellcome Trust Intermediate Fellowship in Tropical Medicine and Public Health (grant # 099992/Z/12/Z) to Dr Standwell Nkhoma. Collection of 2012 samples was supported by the National Institutes of Health, USA through grant # 1U19AIO89683-01 to Professor Terrie Taylor and Dr Miriam Laufer.

## Figures and Tables

**Table 1:** Summary statistics for parasite sampling and basic genetic data.

Year	Sample size	Proportion of MIs	*H*_*e*_	% of identical infections	Multilocus LD (all genotypes)	Multilocus LD (unique genotypes)
2006	85	0.76 ± 0.42	0.37 ± 0.16	10	0.0020	−0.0004
2007	86	0.78 ± 0.41	0.40 ± 0.16	11	0.0080	−0.0004
2008	74	0.69 ± 0.46	0.40 ± 0.16	17	0.0095	−0.0006
2012	50	0.68 ± 0.47	0.32 ± 0.17	13	0.0104	0.0013
2006 and 2007	171	0.77 ± 0.42	0.38 ± 0.15	10	0.0038	0.0009
2008 and 2012	124	0.69 ± 0.46	0.37 ± 0.16	15	0.0059	0.0022

Proportion of MIs is the percentage of patients infected with multiple parasite genotypes. *H*_*e*_ is the expected heterozygosity index. % of identical infections is the percentage of single-genotype infections that share alleles at all the 24 SNPs genotyped. Multilocus LD (all genotypes) is multilocus linkage disequilibrium estimated by including all identical parasite genotypes while multilocus LD (unique genotypes) is that estimated by including single representatives of identical parasite genotypes. Error bars are standard deviations. We observed no multilocus linkage disequilibrium (*p* > 0.05) and no significant change in the proportion of MIs, *H*_*e*_ and % of identical infections over time.

**Table 2:** Variance effective population size of *P. falciparum* in Ndirande, Blantyre, Malawi.

Comparison	ML *N*_*e*_	Pollak *N*_*e*_	Nei/Tajima *N*_*e*_	Jorde/Ryman *N*_*e*_
2006 vs 2007	∞ [70–∞]	73 [22–839]	82 [24–3160]	72 [36–120]
2007 vs 2008	∞ [123–∞]	121 [30–∞]	141 [32–∞]	125 [61–211]
2008 vs 2012	361 [107–∞]	125 [48–346]	141 [52–423]	129 [63–218]

ML *N*_*e*_ is the variance effective population size (*N*_*e*_*V*) estimated using the pseudo-maximum likelihood method. Pollak *N*_*e*_, Nei/Tajima *N*_*e*_ and Jorde/Ryman *N*_*e*_ are the different measures of the variance effective population size estimated using the moments-based temporal methods. Figures in square brackets are 95% confidence intervals for *N*_*e*_*V*.
